# Pluralising Scholarship: Repositioning Doctor of Nursing Practice Faculty Through Boyer's Framework: A Discursive Paper

**DOI:** 10.1111/jan.70577

**Published:** 2026-03-20

**Authors:** Rachel Wangari Kimani

**Affiliations:** ^1^ Binghamton University, Decker College of Nursing and Health Sciences Binghamton New York USA

**Keywords:** academic equity, Boyer's model, DNP faculty, doctoral education, nursing scholarship, practice‐based doctorate, tenure and promotion

## Abstract

**Aim:**

To critically examine the structural exclusion of Doctor of Nursing Practice (DNP)‐prepared faculty from academic advancement and promotion pathways and to propose reforms grounded in Boyer's model of scholarship.

**Background:**

The DNP is a practice‐focused doctorate established in the United States, distinct from the research‐oriented PhD. Similar professional doctorates in the United Kingdom and Australia share the goal of integrating clinical expertise with scholarly inquiry. Despite the rapid growth of DNP programs and the increasing recognition of applied scholarship, many universities continue to privilege traditional research metrics in academic tenure and promotion. This narrow focus on discovery‐based outputs marginalises the contributions of DNP faculty in implementation science, systems leadership, and education.

**Design:**

Discursive position paper.

**Data Sources:**

Analysis of policy reports, faculty promotion guidelines, AACN Essentials, and peer‐reviewed literature on doctoral education, professional doctorates, and academic equity, 2000–2025.

**Implications for Doctoral Education:**

Current academic evaluation systems sustain hierarchical norms that undervalue practice‐based scholarship. This misalignment restricts the career trajectories of DNP‐prepared faculty and constrains nursing's leadership in applied innovation. Reframing scholarly legitimacy through Boyer's model of discovery, integration, application, and teaching enables recognition of diverse expertise without compromising academic rigour.

**Conclusion:**

Fully integrating professional doctorates into academic structures requires deliberate reforms in evaluation frameworks, mentorship programs, and institutional policies. Such changes would advance equity, reflect the realities of modern nursing, and align doctoral education with the evolving needs of healthcare systems.

**Impact:**

This paper contributes to the international discourse on the future of doctoral education by offering a practical model for inclusive faculty advancement. It also advocates adopting pluralistic definitions of scholarship to support diverse academic career paths in nursing.

**No Patient or Public Contribution:**

No patients, service users, caregivers, or members of the public were involved in the development of this discursive paper. The analysis synthesises existing scholarship, policy documents, and theoretical frameworks and does not draw on primary data requiring patient or public involvement.

## 1 Introduction

Despite the substantial growth in Doctor of Nursing Practice (DNP) programs and graduates within the United States, structural impediments continue to hinder their full participation in academic and scholarship roles. As of 2023, more than 430 accredited DNP programs across all 50 states enrolled over 41,800 students (American Association of Colleges of Nursing (AACN) 2024). Nearly 60% of DNP graduates signal a strong interest in academic careers (Beeber et al. 2019; Loomis et al. 2007). However, this expanding pipeline confronts entrenched academic hierarchies, with only 14%–30% of nurse educators holding tenure‐track positions, which constrains their advancement, security, and voice within the academy (National League for Nursing 2024). Vacancy rates among nursing faculty hover between 7% and 9%, with nearly 80% of open positions requiring or preferring a doctoral degree (AACN 2023a; Byrne et al. 2022). These statistics reveal a fundamental misalignment: although DNP‐prepared nurses are prepared and may be willing to teach, academic frameworks remain inadequately equipped to acknowledge or appropriately reward their contributions.

This paper defines practice‐based scholarship as the systematic generation and application of knowledge arising from professional or clinical practice, distinct from discovery‐oriented research, which is typically evaluated through peer‐reviewed publications and competitive grant funding (Sherwood 2024; Wilkes et al. 2013). Practice‐based scholarship synthesises theory, evidence, and reflection to enhance outcomes in clinical care, education, and systems development (AACN 2018; Boyer 1990). Globally, academic promotion and tenure systems have long prioritised research productivity, competitive grants, and peer‐reviewed publications as the highest indicators of scholarly excellence (Avery et al. 2022; Milner et al. 2023). While these measures promote rigour and accountability, they often undervalue applied, practice‐based, and translational scholarship; a challenge seen across professional doctorates in nursing, education, and the health sciences.

Beneath these trends lies a paradox: nursing academia remains structurally misaligned with the very workforce it seeks to sustain. In the United States, while PhD programs in nursing gained prominence in the 1980s as research‐intensive, National Institutes of Health (NIH)‐aligned degree programs, the DNP was introduced in the early 2000s to address pressing needs in clinical leadership, systems improvement, and evidence‐based practice (Ketefian and Redman 2015). Regrettably, unlike the PhD, which benefits from established funding sources and academic prestige, the DNP has frequently encountered difficulties securing a clearly defined position within the academy.

Studies show that evaluation systems frequently undervalue practice‐based work despite its impact on health system outcomes. Udlis and Mancuso (2015) found that nursing leaders recognised DNP faculty contributions to clinical innovation and systems improvement but identified a persistent misalignment between promotion criteria and the forms of scholarship that DNPs typically undertake. Similarly, in the United Kingdom, Avery et al. (2022) reported that clinical faculty encountered barriers related to the availability and definition of research, as well as access to funding. In both contexts, expecting parity with PhD benchmarks without comparable infrastructure or institutional support was seen as inequitable. These findings suggest that the marginalisation of practice‐based scholars likely stems from academic norms rather than a deficit in rigour or impact.

While this critique highlights the structural limitations of conventional metrics, it is also important to recognise why such standards remain central to academic evaluation. Peer‐reviewed publications and competitive grant funding have historically served as key indicators of scholarly excellence because they promote transparency, peer validation, and the cumulative advancement of knowledge (Hug and Aeschbach 2020; Recio‐Saucedo et al. 2022). These benchmarks have played a vital role in maintaining academic rigour and public trust in research (Manley et al. 2013). However, when these measures become the primary or exclusive criteria for advancement, they risk narrowing the definition of scholarship to a single epistemological tradition (Gross and Bergstrom 2019).

Recent scholarship has begun to address these challenges by calling for stronger institutional support to help DNP‐prepared faculty thrive within academia. A growing body of literature now calls for structured mentorship, scholarly writing development, and clearer expectations for faculty productivity (Ea et al. 2021; Hebert and Harding 2024; Vessey et al. 2021). Others advocate expanding the definitions of scholarship to reflect teaching, clinical innovation, and health systems leadership (McCauley et al. 2020; Ramirez et al. 2022). Although valuable, most interventions focus on individual development rather than systemic reform. This paper advances the discourse by examining how conventional promotion and tenure criteria structurally marginalise practice‐based doctoral faculty. Using Boyer's model of scholarship as a conceptual framework, it reframes the issue as one of institutional design rather than personal deficit and proposes actionable strategies to advance equity in faculty evaluation and recognition.

## 2 Structural Barriers and Perceptions of DNP Faculty

Since its introduction in 2004, the DNP degree has undergone significant shifts in its focus, moving away from its original emphasis on advanced clinical practice, health systems leadership, and policy engagement (AACN 2004). Instead of replacing Master's nursing programs, DNP curricula have expanded into educational and leadership domains, diverging from their original intent of preparing a workforce for advanced clinical practice (McCauley et al. 2020). As Muñoz et al. (2023) observe, this expansion blurs lines between practice, pedagogy, and scholarship, causing ongoing ambiguity about the degree's purpose. That ambiguity has tangible consequences: DNP graduates entering academia as terminally prepared faculty often find their qualifications questioned, particularly in comparison to their PhD‐trained peers. These role uncertainties, amplified by inconsistent program structures, reinforce broader institutional patterns of exclusion, affecting hiring, tenure eligibility, and perceptions of scholarly contribution.

The underrepresentation of DNP faculty on tenure‐track positions is well documented, yet it is too often framed as a matter of individual deficit rather than systemic design. Studies consistently point to a confluence of institutional challenges, such as credential bias, poorly defined role expectations, and limited support for practice‐based scholarship that constrain career progression. For example, Fang and Bednash (2017) reported that only 32% of DNP students intended to pursue academic careers, citing low pay, inadequate teaching preparation, and a hostile academic climate. Iwama et al. (2023) and Moran et al. (2025) echoed these concerns, highlighting the institutional invisibility of the DNP role and a lack of mentorship to support scholarly development. These challenges are often compounded by ‘degree‐shaming’ (Bice et al. 2019), in which DNP credentials are perceived as inferior to PhDs, reinforcing academic hierarchies that marginalise practice‐focused expertise.

Although the 2021 AACN Essentials delineate implementation science, systems leadership, and translational research as fundamental components of DNP education (AANC 2021), faculty advancement structures have yet to align accordingly. These practice‐oriented domains are frequently marginalised within faculty progression systems. Further complicating the matter is the persistent lack of role clarity between nurses prepared at the DNP and PhD levels. Despite repeated efforts by AACN to distinguish the objectives, outcomes, and competencies of each degree (AACN 2004, 2010, 2021), role overlap continues to exist, especially in academic environments where both doctoral pathways often compete within the same promotional frameworks (Moran et al. 2025). Consequently, DNP‐prepared faculty are evaluated based on criteria that neither adequately reflect their training nor acknowledge their fundamental contributions to systems enhancement, implementation, and patient‐centered care.

The structural misalignment between academic expectations and practice‐based scholarship is particularly evident in the implementation of DNP student projects. While accreditation standards require these projects to be grounded in evidence‐based practice or quality improvement and include sustainability components (AACN 2023b), implementation often falls disproportionately on clinical sites. As Thornton et al. (2024) report, these systems frequently provide staffing, infrastructure, and oversight without reimbursement, protected time, or input on project design. This matters because it shifts academic deliverables to practice settings that may lack resources or authority, thereby risking project sustainability and compromising the integrity of academic–clinical partnerships. Meanwhile, DNP programs often lack standardised expectations for project scope, mentorship, and evaluation, as noted by Wright et al. (2022), further undermining outcomes and credibility. These tensions highlight the broader disjunction between institutional structures and the applied, collaborative nature of DNP scholarship, leaving faculty and clinical partners alike without the necessary support to fulfil their educational and practice goals.

The contrast with physician workforce models is particularly striking. Physicians in full‐time clinical roles are routinely granted protected time for quality improvement, research, and administrative responsibilities, acknowledging these as integral to their professional scope. In contrast, such structural support remains rare for advanced practice nurses, despite the National Academies of Sciences, Engineering, and Medicine (NASEM) (2021) recognising nursing as ideally positioned to lead in these domains (NASEM 2021). Compounding this disparity, DNP‐prepared faculty are routinely excluded from key research funding mechanisms that drive academic advancement (Wakefield et al. 2021).

As Adler (2025) notes, prestigious programs, such as the Department of Defense's Congressionally Directed Medical Research Program, prohibit nurse practitioners from serving as principal investigators. Many state‐level initiatives, such as the Cancer Prevention and Research Institute of Texas, similarly restrict eligibility to physicians with accredited medical or osteopathic qualifications (Adler 2025). These exclusions persist despite ample evidence that nurse practitioners (NPs), including DNPs, lead impactful research initiatives. As Adler argues, these barriers are not accidental but are, in actuality, rooted in historical gender and professional hierarchies that continue to shape academic norms and funding policies.

Despite systemic barriers, critiques of the DNP frequently misplace blame on the degree's academic rigour or perceived limitations. Some administrators question whether DNP faculty meet methodological standards for tenure, citing inconsistent scholarly output (Nicholes and Dyer 2012; Roush and Tesoro 2018). However, these concerns often ignore the complexity of implementing change in real‐world settings. Practice‐informed scholarship, especially in implementation science and translational research, requires contextual awareness, stakeholder collaboration, and methodological flexibility. As Radzyminski (2023) emphasises, this work is just as rigorous, but of a different kind and equally deserving of academic recognition. Dismissing it through the lens of traditional research hierarchies obscures its relevance to modern healthcare transformation.

Nearly two decades ago, Meleis and Dracup (2005) cautioned that the DNP could fragment the discipline and marginalise its graduates, warning that DNP‐prepared faculty might be excluded from tenure and denied meaningful academic engagement. Their concerns have proven to be prescient. Thornton et al. (2024) report that DNP projects often place disproportionate demands on clinical sites without yielding sustainable or system‐aligned outcomes. Hospital‐based nurse scientists describe how institutions invest significant resources, such as mentorship, infrastructure, and oversight, frequently without reimbursement or input into project design. These challenges are echoed by Dols et al. (2017) and Wright et al. (2022), who highlight gaps in curricular consistency, Quality Improvement (QI) expertise among faculty, and the need for structured writing support. Collectively, these conditions undermine the credibility of DNP scholarship, but they reflect institutional design failures rather than shortcomings of the degree or its graduates.

Although structural barriers persist, the contributions of DNP‐prepared faculty continue to profoundly shape modern healthcare delivery. Their contributions include reducing opioid prescribing (Waszak et al. 2018), expanding access to rural telehealth services, and improving maternal care coordination through nurse‐led care models. In a national study, Kesten et al. (2022) found that DNP‐led scholarship had the greatest impact on care quality, interdisciplinary collaboration, and leadership development. These examples affirm that real‐world, systems‐oriented scholarship is not only effective but essential. The problem is not with the DNP model but with institutional systems that are slow to adapt. As Wright et al. (2022) and AACN essentials maintain, realising the DNP's full potential requires investment in mentorship, faculty development, and evaluation infrastructure aligned with contemporary practice.

These structural misalignments are particularly troubling given current workforce trends. Between 2012–2013 and 2021–2022, U.S. PhD nursing program enrollment decreased by 14.5%, with graduation rates remaining flat (Halabicky et al. 2024), and this trend is expected to continue. This data challenges the notion that supporting DNP faculty undermines the PhD pipeline. On the contrary, broadening academic pathways through inclusive doctoral models is critical to rebuilding nursing's scholarly workforce. Clinging to narrow definitions of academic merit only exacerbates faculty shortages and weakens the discipline's future.

Non‐tenure‐track roles are often promoted as viable alternatives for DNP faculty, yet these positions are typically under‐resourced and offer limited opportunities for advancement, scholarship, or institutional voice. The result is a two‐tier faculty system in which DNPs are expected to teach and lead without the protections or recognition granted to tenure‐track peers. As Ashcraft et al. (2021) note, tenure safeguards the conditions necessary for genuine scholarship by protecting academic freedom and allowing faculty to pursue innovative or even controversial lines of inquiry.

Thus, excluding DNP faculty from these protections marginalises their contributions and reinforces their peripheral status within the academy. Lacking clear pathways to advancement, many are disincentivised from engaging in sustained inquiry and leadership, ultimately undermining nursing's capacity for translational science and innovation. Further practice‐based faculty exclusion weakens the academic mission by narrowing the scope of what constitutes legitimate scholarship.

### 2.1 Lived Examples: Navigating Faculty Hiring as a DNP

Experiences of DNP‐prepared faculty often illuminate how institutional hierarchies, rather than individual performance, shape career progression. Despite mentorship, peer‐reviewed publications, and applied scholarship, DNP faculty frequently encounter gatekeeping in hiring and promotion processes that continue to privilege the PhD as the default credential for academic legitimacy.

This pattern is well‐documented in the literature. In a recent qualitative study, Muñoz et al. (2023) found that DNP‐prepared faculty often pursued PhDs not for additional research training but to meet institutional expectations rooted in historical credential hierarchies. Similarly, Berg and Hicks (2024) describe how the subtle devaluation of practice‐based scholarship fosters a cycle of overcompensation and credential escalation. Dunlap (2024) reflective account underscores this tension: hired for translational expertise, she was advised to pursue a PhD to achieve ‘true’ scholarly status. Her experience, echoed by others, exemplifies the persistent conflation of academic merit with discovery‐oriented research and a doctoral title rather than with demonstrable impact.

From my own experience navigating faculty hiring as a DNP‐prepared educator, these dynamics are strikingly familiar. Despite a publication and leadership record comparable to that of many early‐career PhD‐prepared faculty, legitimacy often remained tied to the credential rather than to demonstrated scholarly contribution. Pursuing a PhD thus became less an act of new intellectual pursuit and more a pragmatic response to institutional expectations and a means of negotiating entry into structures still resistant to recognising applied, pedagogical, or translational work as academically rigorous. Such experiences reveal how the academy's insistence on a single pathway to legitimacy not only marginalises practice‐based scholars but also narrows the intellectual and social reach of nursing itself.

## 3 Reframing Scholarship Through Boyer's Model

Reimagining academic advancement for DNP‐prepared nurses requires challenging the narrow standards of scholarly excellence embedded in tenure and promotion systems. These frameworks continue to prioritise traditional discovery outputs, such as randomised controlled trials or NIH‐funded research, while overlooking contributions in teaching, policy, systems leadership, and clinical innovation. Ironically, the forms of scholarship most aligned with DNP education are often least valued, exposing a persistent disconnect between institutional review practices and the mission of practice‐based nursing education.

**FIGURE 1:**
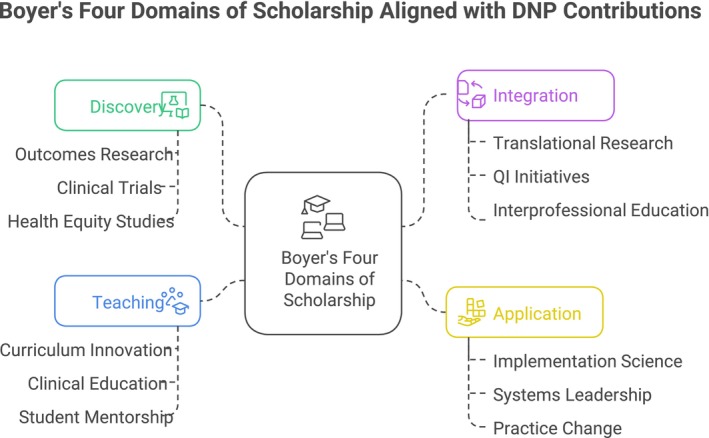
Boyer's four domains of scholarship aligned with DNP faculty contributions. This framework legitimises practice‐based, translational, and pedagogical scholarship as academically rigorous and essential to the transformation of health systems.

First introduced in 1990, Boyer's model of scholarship offers a broader and more inclusive framework for evaluating academic contributions (Boyer 1990). Instead of focusing solely on research discovery, Boyer identified four interconnected domains: discovery, integration, application, and teaching. These categories better capture the diverse, practice‐informed knowledge that defines professional fields, such as nursing, particularly in doctoral‐prepared roles (Boyer et al. 2015). Figure 1 illustrates how these four domains align with DNP faculty work, connecting outcomes research, quality‐improvement initiatives, systems leadership, and curriculum innovation within a unified model of scholarly contribution.

DNP curricula are intentionally aligned with these broader dimensions of scholarship. For instance, a DNP faculty member might evaluate a statewide maternal health intervention, integrate social determinants data into practice, co‐lead clinical trials for a new care model, or innovate methods for teaching advanced practice nursing. These activities exemplify not only academic rigour but also real‐world relevance, yet they often fall outside what tenure committees traditionally consider scholarship.

**FIGURE 2:**
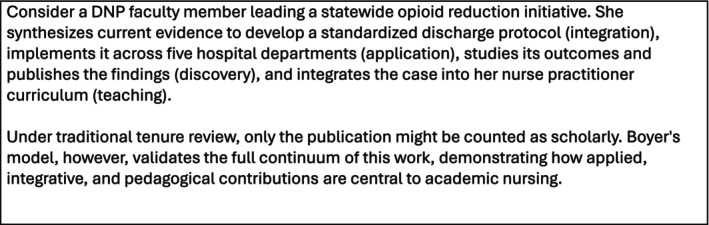
Case example: scholarship across Boyer's domains.

Figure 2 deepens this point through a case example: a DNP faculty member spearheads an opioid reduction initiative that generates outcomes research (discovery), protocol development (integration), systems‐level implementation (application), and curricular innovation (teaching). Under conventional review systems, only the publication might ‘count’. But through Boyer's lens, each component is an expression of rigorous, impactful scholarship.

Some institutions have begun operationalising this broader framework. At Rush University, faculty organise scholarly portfolios around Boyer's domains, advancing work in health equity and disability care. At the University of Texas Health Science Center at San Antonio, DNP and PhD faculty co‐lead community‐based behavioural health projects that span research, education, and practice (Adler and Sickora 2023). These models demonstrate how Boyer's framework can support both faculty development and translational impact.

Despite promising examples, most nursing and health sciences institutions in the United States continue to undervalue applied and integrative scholarship in faculty advancement. National data from U.S. medical schools indicate that while 90% of medical schools maintain tenure systems, only 44% of new basic science hires and just 10% of new clinical faculty are placed on tenure‐eligible tracks (Mallon and Cox 2024). Though drawn from medicine, these trends mirror what DNP‐prepared faculty face in nursing, where appointments often default to clinical or instructional roles without a pathway to tenure. This reveals a deeper contradiction between what institutions profess to value and how they structure academic advancement. As Clark et al. (2020) argue, practice‐based contributions are not dismissed for lack of rigour but rather because they fall outside outdated evaluation frameworks that lack clear criteria, mentorship, and long‐term planning.

These challenges are not limited to the United States. In the United Kingdom, Trusson et al. (2019) found that nurses, midwives, and allied health professionals (NMAHPs) pursuing clinical academic careers face persistent barriers, including limited post‐PhD opportunities, a lack of integrated academic–practice pathways, and the undervaluation of applied or educational scholarship. A 10‐year review by the UK's National Institute for Health and Care Research (NIHR) found that nurses and midwives receive less research funding than other healthcare professionals, particularly at the doctoral level, raising concerns about equity and long‐term career progression.

Australia offers partial progress. Faculty practice models aim to bridge the gap between theory and clinical work (Currie et al. 2022). For example, Fowler et al. (2017) found that Australian nursing academics who participate in faculty clinical practice report enhanced teaching, increased confidence, and improved clinical‐academic collaboration. However, these models remain unevenly implemented due to time constraints, ambiguous institutional expectations, and inconsistent clinical support.

In Canada, Boamah et al. (2021) highlight how research‐centric promotion criteria, uncompetitive salaries, and high workloads contribute to a persistent faculty shortage, despite growing demand for nursing educators. These factors disproportionately affect faculty whose scholarly work centers on teaching, systems leadership, or clinical innovation. Although some Canadian institutions have signalled a move towards incorporating Boyer's domains, there is limited peer‐reviewed analysis of how widely or consistently such frameworks are implemented. A persistent tension spans institutional contexts: the scholarship most deeply connected to nursing's societal impact often remains least visible within promotion systems still structured by linear, traditional models of academic merit.

While Thamm et al. (2025) emphasise the urgent need to support PhD‐prepared nurses in clinical academic roles, their analysis also highlights the structural tensions that hinder practice‐based scholarship more broadly. They argue that the greatest benefit to patients lies not solely in generating new knowledge but in implementing existing evidence‐based guidelines and clinical innovations effectively. This perspective aligns closely with Boyer's domains of application and integration; areas where DNP‐prepared faculty are uniquely positioned to lead. With expertise in systems leadership, quality improvement, and evidence translation, DNPs are well‐equipped to bridge the gap between research and real‐world care. Rather than reinforcing hierarchies, institutions should promote collaborative faculty models that recognise diverse forms of scholarly contribution. Embedding DNP–PhD partnerships on equal footing would strengthen nursing's academic mission and bring Boyer's inclusive vision of scholarship to life.

Still, critics caution that adopting Boyer's model is not a panacea. Crow et al. (2018) found that institutional efforts to implement Boyer's framework often result in ‘widespread but shallow’ change, where policies may reflect the model. However, faculty norms and reward structures continue to prioritise traditional research. This tension between stated values and entrenched practices leads to ongoing uncertainty about how applied scholarship should be defined, documented, and evaluated. Similarly, Hofmeyer et al. (2007) note the lack of transparent metrics for evaluating integration and application domains, which can discourage faculty from pursuing these forms of scholarship. These second‐generation challenges of implementing without cultural change highlight the difficulty of embedding applied scholarship in the health sciences, where rhetorical support is often not backed by evaluative legitimacy.

Recent work by Dunlap et al. (2024) reinforces the urgent need for scholarship standards that align with the strengths of DNP‐prepared faculty. Their synthesis of promotion criteria across US institutions found that while Boyer's framework is often referenced, expectations for DNP scholarship remain vague and inconsistently applied. The authors advocate shifting the evaluative focus from traditional research outputs to the impact of scholarly work, including policy influence, quality‐improvement outcomes, and clinical innovation. They also provide actionable tools, including templates and evaluation metrics, that institutions can adopt to operationalise Boyer's domains in a way that validates DNP contributions. Incorporating these standards across faculty review processes would not only support equity but also expand the collective impact of nursing scholarship.

To fully realise Boyer's model, institutions must move beyond rhetorical support and invest in structural reforms. These include creating portfolio‐based promotion criteria that value clinical innovation, curriculum design, and systems leadership; providing mentorship tailored to practice‐based faculty; and developing transparent metrics for evaluating integrative and applied scholarship. Without such mechanisms, Boyer's model risks becoming symbolic rather than transformative. A reimagined system aligned with the full spectrum of scholarly work will not only promote equity for DNP faculty but also strengthen nursing's collective impact on health systems, education, and patient care.

## 4 Discussion

DNP programs have experienced rapid expansion over the past decade. Nevertheless, despite the escalating demand for nursing faculty, DNP‐prepared faculty members remain structurally marginalised within academic institutions (Bloch and Glasgow 2023). This marginalisation does not reflect their individual capabilities but is instead a consequence of persistent institutional frameworks that continue to prioritise a narrow, discovery‐oriented conception of scholarship. Faculty members whose expertise lies in systems leadership, quality improvement, and translational care are frequently expected to undertake substantial academic responsibilities while being denied access to tenure protections and scholarly legitimacy.

The consequences are both systemic and personal. DNP faculty are hired into critical teaching and clinical leadership roles, yet they are frequently excluded from institutional governance, protected time, and research infrastructure (Van Dongen et al. 2024). They are expected to design curricula, mentor advanced practice students, and lead change initiatives, but do so under advancement structures that prioritise peer‐reviewed publications and grant‐funded research over the impact of their practice. The result is a tiered faculty system, shaped by credential bias and outdated review criteria, that marginalises applied scholarship and restricts career mobility (Ashcraft et al. 2021).

An unsustainable triad of expectations exacerbates these inequities: many DNP faculty are required to maintain clinical practice, teach full course loads, and produce scholarship without the support, time, or recognition afforded to PhD‐trained colleagues. This threefold burden fosters burnout and churn, undercutting continuity in nursing education and weakening the pipeline of practice‐based academic leaders (Melnyk et al. 2023; Zangaro et al. 2023).

However, the solution is not to assimilate DNP faculty into legacy research norms, but to evolve our conception of scholarly excellence itself. Boyer's model of scholarship offers a more expansive, practice‐aligned alternative (Ramirez et al. 2022). Its four domains (discovery, integration, application, and teaching) reflect the full spectrum of academic contributions that DNPs are uniquely positioned to make. From clinical innovation to systems redesign to pedagogy and policy engagement, DNP faculty are actively producing meaningful scholarship across these domains.

Nevertheless, institutional policies have not kept pace with developments. Tenure and promotion frameworks persist in emphasising narrowly defined outputs, such as data‐driven research, R01 grants, and impact factors, while disregarding the forms of knowledge creation most essential to contemporary health systems. Even the DNP project, which should serve as a culmination of translational inquiry, is frequently limited to narrow quality improvement formats with constrained scope and visibility (Hickey and Giardino 2023).

Boyer's model should transcend mere citation in strategic plans and must be actively implemented through intentional reforms in evaluation standards, mentorship frameworks, funding mechanisms, and academic culture (O'Brien et al. 2019). When adequately resourced and acknowledged, translational scholarship has a significant impact on patient outcomes and system performance (Trautman et al. 2018). The central question is no longer whether DNP faculty are part of the academy; they already are. The question is whether institutions are willing to evolve to ensure DNPs can thrive.

## 5 Policy and Structural Recommendations

Repositioning DNP‐prepared faculty needs more than rhetoric; it requires structural change. To align policies with their scholarly contributions, this paper outlines six reforms. These target barriers and norms that limit the visibility, evaluation, and advancement of practice‐based scholarship.

Recent institutional exemplars demonstrate that progress is both possible and measurable. In the United States, Rush University College of Nursing integrated practice‐based scholarship into its academic structure by creating a Department of Academic Practice Nursing (DAPN) and a Practice Scholarship Advisor role, resulting in measurable increases in scholarly dissemination (Cygan and Moss 2025). A systematic scoping review identified similar initiatives nationwide, including PhD‐DNP collaborations and mentorship initiatives, which improved promotion outcomes among practice‐based faculty (Dunlap et al. 2025; Pinto et al. 2021). Comparable reforms have also been adopted across academic health sciences, where medical schools have revised promotion criteria to recognise educational and team‐based scholarship (Chang et al. 2023; Goldstein et al. 2018). In the United Kingdom, nursing institutions have also advanced comparable efforts through clinical academic pathways and practice‐based research collaborations that embed scholarship within care delivery and education (Carrick‐Sen et al. 2019; Gerrish and Chapman 2017). Collectively, these initiatives show that diverse forms of scholarship can be integrated into academic systems worldwide. Table 1 summarises recommended actions and responsible stakeholders.

**TABLE 1:** Recommendation of institutional reforms to advance and recognise DNP faculty scholarship.

Priority area	Key actions	Responsible stakeholders
Redefine scholarship standards	Embed Boyer's four domains (discovery, integration, application, and teaching) into tenure and promotion criteria. Use national guidance (e.g., AACN) to develop evaluative tools for practice‐based scholarship	Faculty Affairs Committees, Deans, Accrediting Bodies
Stabilise faculty roles and reduce burnout	Formalise promotion pathways and workload protections for teaching‐ and clinically intensive faculty. Provide protected time and equitable distribution of service expectations	Provosts, Department Chairs, Human Resources
Strengthen mentorship and development	Implement structured mentorship programs to support promotion readiness and impact documentation for practice‐based scholars	Office of Faculty Development, Promotion & Tenure Committee
Expand the DNP scholarly agenda	Broaden DNP project formats to include policy, pedagogy, and systems‐level innovation. Support DNP–PhD collaborations to bridge research and practice	Graduate Curriculum Committees, Research Directors
Track and address equity by degree type	Monitor and report promotion, compensation, and leadership metrics by doctoral pathway to identify and address structural disparities	Institutional Research Offices, DEIA Committees
Foster a culture of scholarly inclusion	Embed recognition of applied and translational scholarship into awards, leadership appointments, and professional development programs	Academic Affairs, Awards and Recognition Committees

*Note:* This table outlines six priority areas and associated actions that institutions can adopt to support the advancement and recognition of DNP‐prepared faculty, particularly in academic systems still oriented towards traditional research metrics. Together, these policies advocate for the intentional redesign of evaluation criteria, mentorship structures, and institutional cultures to ensure that all forms of scholarship, discovery, integration, application, and teaching are valued equitably across global higher education contexts.

## 6 Conclusion

The marginalisation of DNP‐prepared faculty is not attributable to a transitional phase, but rather to an inherent flaw in the system's design. These clinicians‐turned‐scholars are entrusted with the responsibility of advancing nursing practice; however, they are subjected to evaluation models that fail to recognise their expertise. They are tasked with teaching, leading, and innovating, yet lack the scholarly infrastructure needed to establish legitimacy or sustain their endeavours.

This paper reaffirms the importance of Boyer's model of scholarship, which expands rather than limits the definition of scholarly excellence. Boyer's framework provides a practical way to move forward, as long as institutions make structural changes to apply it consistently and fairly. From recruitment to assessment, and from mentorship to project development, schools of nursing must foster academic environments that support different types of doctoral scholarship.

DNP faculty are integral to academic nursing, not just supportive roles; they play a key part in transforming health systems and preparing practice‐ready clinicians. Recognising and resourcing their contributions is not merely a gesture of inclusion; it is a strategic imperative. The question now is whether the nursing academy will rise to meet this moment or continue to build walls around the very scholars most equipped to lead change.

## Author Contributions

The author conceptualised and wrote the manuscript.

## Funding

The author has nothing to report.

## Ethics Statement

The author has nothing to report.

## Conflicts of Interest

The author declares no conflicts of interest.

## Data Availability

The author has nothing to report.
